# Supplementary material for: Soft elasticity optimises dissipation in 3D-printed liquid crystal elastomers
Source: Nat Commun. 2021 Nov 18;12:6677. doi: 10.1038/s41467-021-27013-0 (PMC8602646; doi:10.1038/s41467-021-27013-0)
Supplement: Supplementary file 1 — Supplementary Information [file 41467_2021_27013_MOESM1_ESM.pdf]

Supplementary Information for Optimizing dissipation through the soft-elasticity of 3D printed liquid crystal elastomers.

D. Mistry[1,2]*, N. Traugutt[1], B. Sanborn[3], R. Volpe[1], Lillian Chatham[4], Risheng Zhou[1], B Song[3], Kai Yu[1], K. Long[3], C. Yakacki[1]*

[1]Department of Mechanical Engineering, University of Colorado, Denver, Denver, CO, 80204, USA.
[2]Present address: School of Physics and Astronomy, University of Leeds, Leeds, LS2 9JT, UK.
[2]Materials and Failure Modeling Department, Sandia National Laboratories, Albuquerque, NM, 87123, USA
[4]Impressio Inc., 12635 E. Montview Blvd, Suite 214, Aurora, CO 80045, USA

a

b

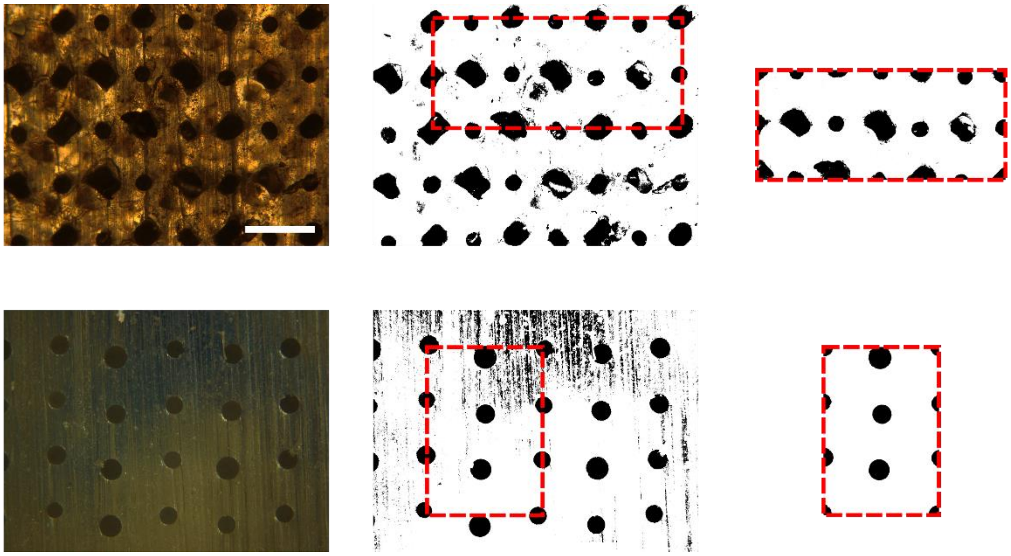

Supplementary Figure 1 Porosity of DIW-printed devices The DIW process trapped channels of air, parallel to the print direction inside printed devices. Thin example slices of the plane perpendicular to the print direction for the printed LCEs (**a**) and BPA-ink elastomer (**b**) are shown, viewed via reflection microscopy. The black regions are slices of the porous channels. By processing these images to separate air and elastomer regions, and defining a representative unit cell, approximation of the devices' porosity could be made. The LCE (BPA-ink elastomer) was calculated to have a porosity of 18 % (9 %). Bar is 1 mm.

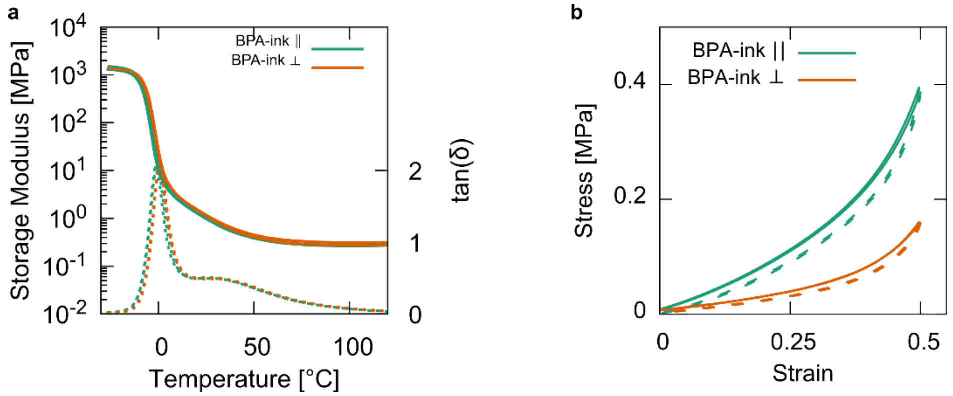

Supplementary Figure 2 Isotropy of BPA-ink elastomer. a DMA temperature-sweeps and, **b**, quasi-static compression tests (10^{-4} s^{-1}) of the 3D printed BPA-ink elastomer devices. In both cases, samples were tested for strains applied parallel and perpendicular to the printed orientation. In **a,** the material's behavior was identical. In **b** the parallel sample behaved stiffer, however the character of the load curves is the same. The figures confirm the fundamental isotropy of the BPA-ink elastomer with respect to printed orientation.

3 EDDT

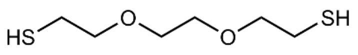

4 BHT

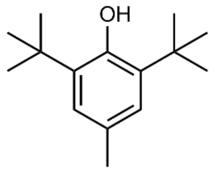

5 HHMP

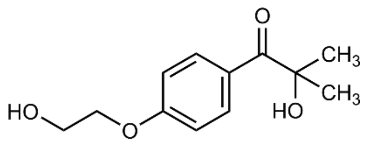

6 PMDETA

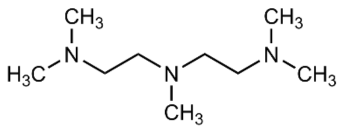

7 PETMP

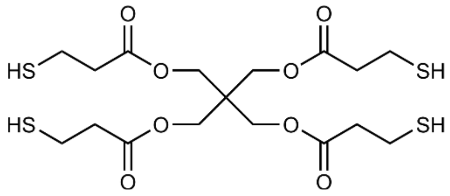

Supplementary Figure 3 Structures of chemicals used in this work. Additional structures of the liquid crystalline and non-liquid crystalline diacrylate monomers also used are shown in figure 2a.

Supplementary Table 1 Drop heights and equivalent parameters of 2 kg drop tests.

Drop height, m	Impact speed, m s^{-1}	Initial nominal strain rate, s^{-1}	Impact energy density, J cm^{-3}
0.25	2.1	270	4.0
0.50	2.9	380	8.0
0.75	3.6	470	12.0
1.00	4.2	540	16.0

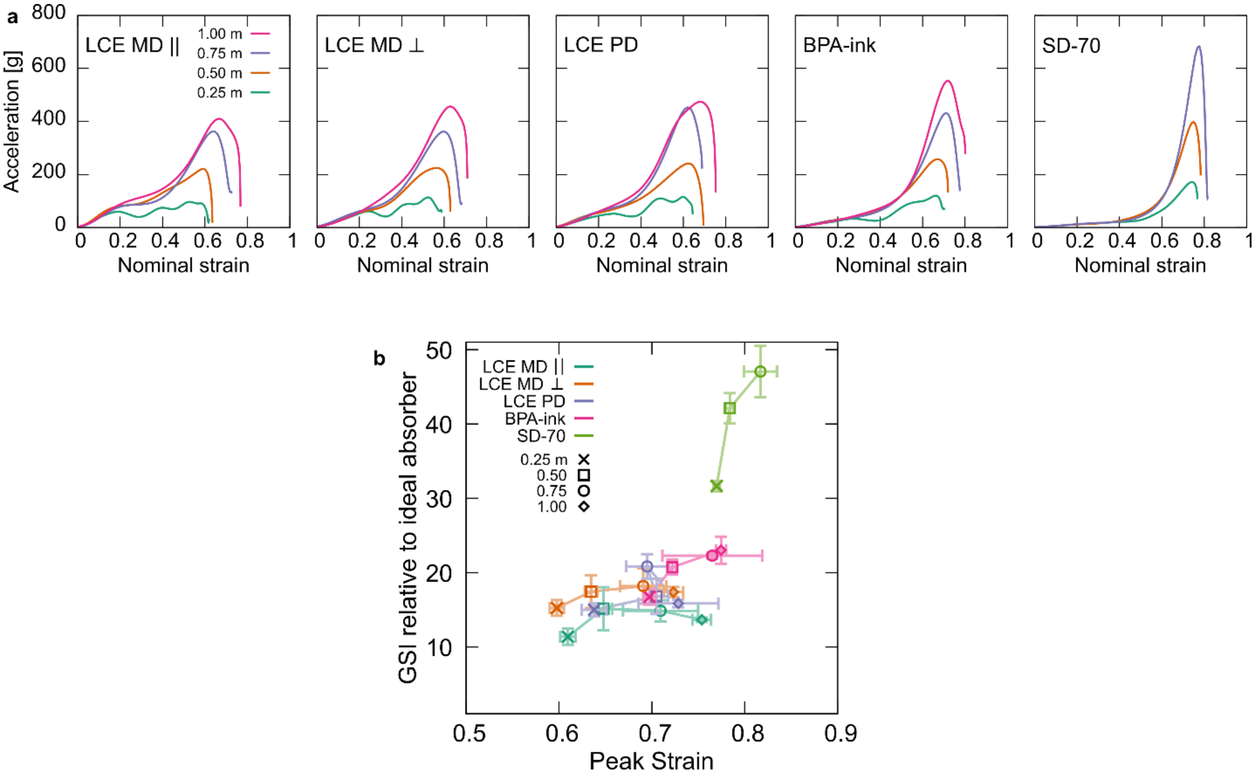

Supplementary Figure 4 a,b Drop test results from figures 4b 4c with the addition of data for LCE MD ⊥ and LCE PD.

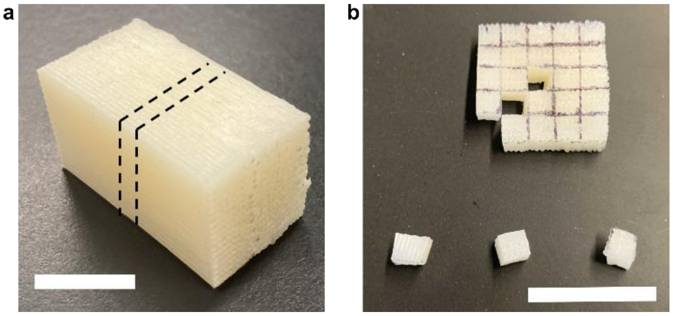

Supplementary Figure 5 Photographs of 20x20x37 mm sample of DIW printed LCE, used to verify complete crosslinking of printed devices. Three 2 mm thick central slices of material, like those illustrated in **a**, were cut from the bulk of device and from those three 4 x 4 x 2 mm^3 cuboids were cut as shown in **b**. Each cuboid was subjectedto gel fraction tests as described in the main methods. Scale bars 20 mm.

Kolsky bar experimental methods

In this study, a Kolsky compression bar was employed to measure the dynamic compressive response of the LCE and BPA over a strain rate range of 800 to 3000 s^{-1}. Several experimental challenges have exist when testing soft materials with a Kolsky bar, including specimen dynamic force equilibrium, constancy of strain rate, interfacial friction, and lateral inertia effect. These effects have been reviewed in detail by Song and Chen (Song 2005).[30] In general, the Kolsky bar needs to possess relatively low mechanical impedance for soft material characterization. Appropriate pulse shaping is required for achieving stress equilibrium and constant strain rate which consequently minimizes lateral inertia effect. The Kolsky compression bar used in this study consisted of incident and transmission bars that were both 3.66 m long, diameter of 25.4 mm, and made of 7075-T6 aluminum. Striker bars were made of the same material and were typically 0.61 to 1.07 m in length. Copper pulse shapers varied in size but ranged between 10 to 13.5 mm in diameter and 1.57 to 3.2 mm in thickness.

In a typical Kolsky bar test, it is estimated that the stress wave needs at least three round trips inside the specimen to achieve stress equilibrium. However, the actual stress equilibrium state during dynamic loading needs to be experimentally verified. Since dynamic force equilibration cannot be precisely verified with the measured strain gage signals[30], quartz crystal force transducers (QCFT) were embedded at the ends of the Kolsky compression bar[31] to directly measure the forces at both ends of the specimen during dynamic loading. A typical set of QCFT signals at both ends of the specimen is shown in Supplementary Figure 6 for a 3.2 mm thick by 11.5 mm diameter polydomain LCE disk. Later specimen geometries were 2 mm thick (and typically 5x5 mm square cross-sections), which causes stress equilibrium to occur earlier. Hence, the 3.1 mm thick specimen in Supplementary Figure 6 represents the slowest equilibrating case in this work. For this test, the pulse shaper was a copper disk with a thickness of 3.1 mm and 11.9 mm diameter. The striker bar was 1.07 m long and was fired with a speed of approximately 10 m/s. As shown in Supplementary Figure 6, the force histories at both ends of the specimen nearly overlapped except for the first 40 microseconds when the specimen was in a “ring up” process to stress equilibrium. The strain and strain rate histories were calculated with the conventional data reduction process and are shown in Supplementary Figure 7. With application of pulse shaping, the strain rate reached a near-constancy after 50 microseconds. Combining Supplementary Figures 6 and 7, the specimen achieved both stress equilibrium and constant strain rate after 50 microseconds of initial loading, which corresponds to 5% strain. This means the resultant stress-strain curve is valid when specimen strain is larger than 5%, which is before the plateau and densitification occurred (the region of interest (ROI) in this study).

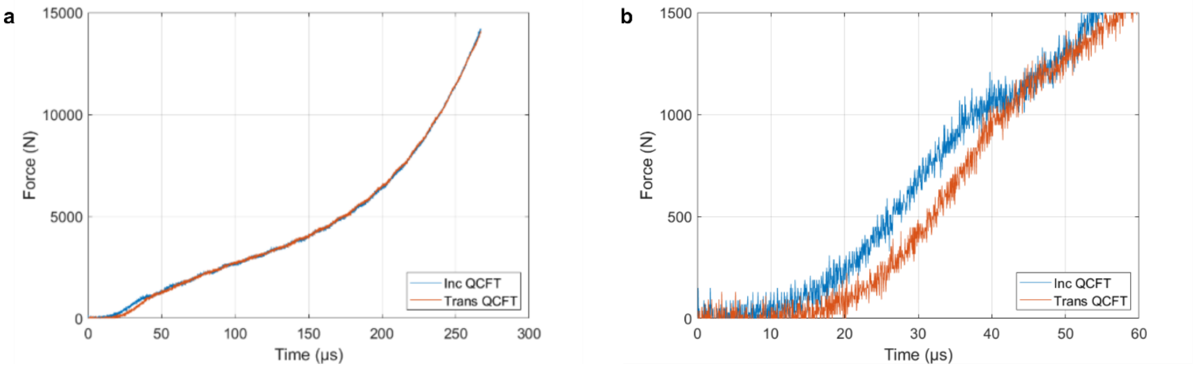

Supplementary Figure 6 Quartz crystal force transducer output from a Kolsky bar test on solid LCE polymer. **a** Force history during entire loading duration, and **b**, zoomed portion showing the response during equilibration. The stress became equilibrated after a time of approximately 45 µs, fulfilling one requirement of a valid Kolsky bar experiment

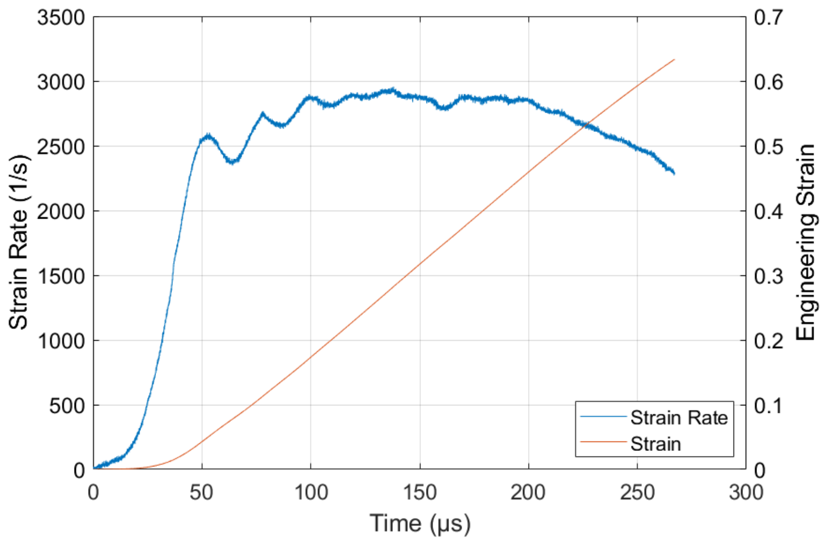

Supplementary Figure 7 Engineering strain rate and strain histories of LCE polymer. This plot shows that the specimen reached constant strain rate after a ramp time of approximately 50 µs.

The constant strain rate in the specimen also minimizes the lateral inertia effect in the specimen. The lateral inertial results in additional axial stress component, which was calculated by Warren and Forrestal,[32]

$$\sigma = \frac{3\rho a^2}{16(1-\varepsilon_x)^3}\dot{\varepsilon_x}^2 + \frac{3\rho a^2}{8(1-\varepsilon_x)^2}\ddot{\varepsilon_x},$$

where ε_x is the specimen strain, $\dot{\varepsilon}_x$ is the specimen strain rate, $\ddot{\varepsilon_x}$ is the specimen strain acceleration, ρ is the density, and a is the radius of the circular specimen. Though our specimens had a small amount of

porosity and a square cross-section, we can use this equation to calculate the approximate magnitude of the radial inertia stress. When the specimen is subjected to constant strain rate, the second term in the equation becomes zero. When a LCE specimen with a density, ρ = 1250 kg/m^3, is subjected to a maximum strain, ε_x = 0.65, at a constant strain rate, $\dot{\varepsilon}_x$=3000 s^{-1}, the additional axial stress generated by the lateral inertia was calculated as approximately 0.2 MPa, which is negligible (0.12%) to the actual axial stress (160 MPa).

Finally, interfacial friction between the specimen and bar ends may lead to higher measured stress due to the change of stress state in the sample from uniaxial to triaxl stress state. Appropriate lubrication of the interface between the specimen and bar ends is required to reduce the interfacial friction. The selection of lubricant depends on both specimen and bar materials. Song et al. 2009 demonstrated the effect of different lubricants on an alumina-filled epoxy in a Kolsky bar experiment, which showed a 25% reduction in the measured plateau stress with proper lubrication compared to a dry interface.[33] We used this same approach in our experiments, which were lubricated using Krytox (GPL-105) fluorinated oil.
